# Role of CBL Mutations in Cancer and Non-Malignant Phenotype

**DOI:** 10.3390/cancers14030839

**Published:** 2022-02-08

**Authors:** Davide Leardini, Daria Messelodi, Edoardo Muratore, Francesco Baccelli, Salvatore N. Bertuccio, Laura Anselmi, Andrea Pession, Riccardo Masetti

**Affiliations:** 1 Pediatric Oncology and Hematology Unit “Lalla Seràgnoli”, IRCCS Azienda Ospedaliero-Universitaria di Bologna, 40138 Bologna, Italy; davide.leardini3@studio.unibo.it (D.L.); daria.messelodi2@unibo.it (D.M.); francesco.baccelli2@studio.unibo.it (F.B.); salvatore.bertuccio2@unibo.it (S.N.B.); laura.anselmi4@unibo.it (L.A.); riccardo.masetti5@unibo.it (R.M.); 2 Department of Medical and Surgical Sciences, University of Bologna, 40138 Bologna, Italy; 3 Pediatric Unit, IRCCS Azienda Ospedaliero-Universitaria di Bologna, 40138 Bologna, Italy; andrea.pession@unibo.it

**Keywords:** CBL, CBL syndrome, cancer predisposition, JMML, pediatric oncology

## Abstract

**Simple Summary:**

*CBL* mutations are progressively being described as involved in different clinical manifestations. Somatic *CBL* mutations can be found in different type of cancer. The clinical spectrum of germline mutations configures the so-called CBL syndrome, a cancer-predisposing condition that includes multisystemic involvement characterized by variable phenotypic expression and expressivity. In this review we provide an up-to-date review of the clinical manifestation of *CBL* mutations and of the molecular mechanisms in which CBL exerts its pathogenic role.

**Abstract:**

CBL plays a key role in different cell pathways, mainly related to cancer onset and progression, hematopoietic development and T cell receptor regulation. Somatic *CBL* mutations have been reported in a variety of malignancies, ranging from acute myeloid leukemia to lung cancer. Growing evidence have defined the clinical spectrum of germline *CBL* mutations configuring the so-called CBL syndrome; a cancer-predisposing condition that also includes multisystemic involvement characterized by variable phenotypic expression and expressivity. This review provides a comprehensive overview of the molecular mechanisms in which CBL exerts its function and describes the clinical manifestation of *CBL* mutations in humans.

## 1. Introduction

Over the last years, large-scale genomic studies have uncovered a genetic predisposition in a large variety of cancers, especially in children [1]. A recent study based on The Cancer Genome Atlas (TCGA) data set reported that approximately 8% of adult patients with cancer have a germline predisposing mutation [2]. A similar prevalence of germline mutations in cancer-predisposing genes has been identified in children and adolescents with cancer [3]. In addition to providing important insights into diagnostic and prognostic information, the evaluation of non-tumor or germline material has provided novel information on previously unrecognized non-malignant clinical spectrum [4,5,6,7,8]. Casitas B lineage lymphoma (*CBL*) is a multifunctional gene codifying for a class of adaptor proteins (CBL family proteins) implicated in the regulation of signal transduction in many physiological and pathological processes. Particularly, CBL has been reported to play a role in different well-known cell pathways, many of those are related to cancer onset and progression, hematopoietic development and T cell receptor regulation. Growing evidence have defined the clinical spectrum of germline *CBL* mutations configuring the so-called CBL syndrome, a cancer-predisposing condition that has been shown to also include multisystemic diseases. This review aims to characterize the molecular mechanisms in which CBL exerts its function and to provide an overall focus on clinical manifestation of *CBL* mutations in humans.

## 2. Structure and Function of CBL

**Figure 1:**
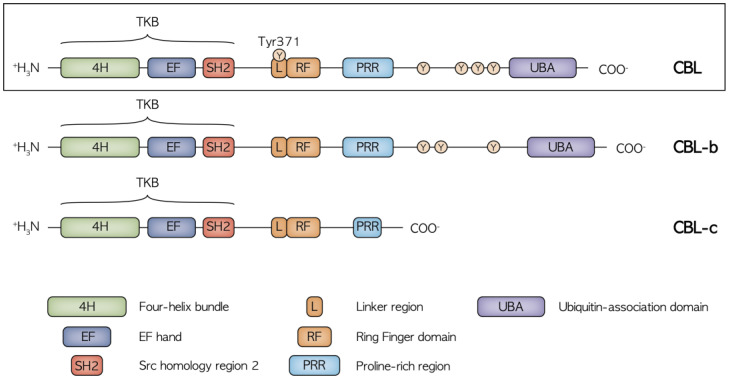
Structure of the CBL, CBL-b and CBL-c proteins with the relative functional domains and main phosphorylation sites. CBL and CBL-b are structurally related while CBL-c lacks the C terminal domain, including the UBA region and the proline and tyrosine rich region, mediating the binding to the SH2 and SH3 domain-containing proteins. The highly conserved N terminal region with the TKB, Linker and RF domains is crucial in the regulation of receptor and non-receptor tyrosine kinase activity.

CBL proteins are included in a highly conserved family of RING finger (RF) ubiquitin ligases (E3s). Three CBL member proteins have been described: CBL, CBL-b and CBL-c [9]. CBL and CBL-b are structurally more related, while CBL-c differs by lacking the C-terminal domains. The structure of CBL protein comprises an N-terminal tyrosine kinase binding (TKB) domain followed by a linker region, a RING domain, a proline-rich region (PRR), a C-terminal phosphorylation site and a ubiquitin-association domain (Figure 1).

The main known function of CBL proteins is the regulation of receptor and non-receptor tyrosine kinases (RTKs and non-RTKs, respectively), which is implicated in the transduction of signals from immune receptors (T-cell receptor, B-cell receptors and Fc receptors). It has been demonstrated that CBL proteins negatively regulate RTKs and non-RTKs proteins through their ubiquitination and degradation [9,10], mediating the transfer of ubiquitin from E2 ubiquitin ligase to specific substrates. The CBL protein normally exists in the cytosol in an inactive state where the catalytic RF domain is masked by the N-terminal TKB domain. More precisely, the E3 activity tightly depends on a conserved link-helix region (LHR) tyrosine (Tyr371), that in normal conditions, is anchored to the TKB domain and constrains the RING domain in an inactive conformation. When stimulated, CBL is recruited to an activated RTK, leading to extensive tyrosine phosphorylation including Tyr371, which induces conformational changes to activate its E3 activity. This process exposes the RF domain allowing increased E2 binding to the CBL protein in closer proximity to the RTK, facilitating the transfer of ubiquitin from the E2 to lysine residues on the RTK. The ubiquitinated RTK then traffics through the endocytic compartments to the lysosome where it is degraded [11]. CBL and CBL-b share additional areas of homology in the C-terminal half of the proteins, including a ubiquitin-association (UBA) domain and more extensive proline and tyrosine-rich regions, which can be phosphorylated and mediate interactions with Src homology region 2 and 3 (SH2 and SH3) domain-containing proteins including GRB2, CD2AP/CIN85, Cool/Pix and p85 subunit of PI3K [12]. These domains play a critical role in CBL adaptor function. CBL inhibition occurs through dephosphorylation mediated by Src-homology-2-containing phosphatase-1 (SH1P) or ubiquitylation mediated by CBL itself or by the HECT-type E3 ligases atrophin-1-interacting protein-4 (AIP4)/Itch or NEDD4 [13].

## 3. The Role of CBL in Signaling Pathway Modulation

**Figure 2:**
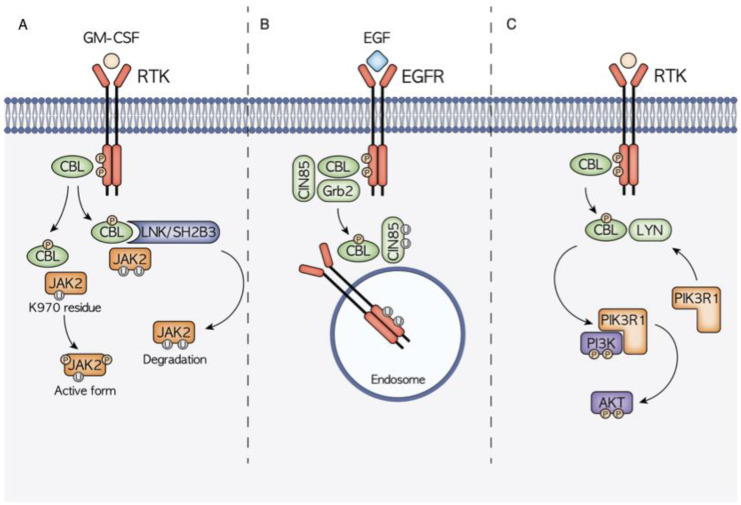
Schematic representation of the three main molecular mechanisms of CBL-mediated signal transduction modulation. (**A**) CBL is hypothesized to have a dual role in JAK2 signaling. On one hand, thanks to the interaction with the mediator LNK, CBL controls the ubiquitination and consequent degradation of JAK2, on the other hand it has been highlighted how the CBL-mediated ubiquitination at the JAK2 K970 residue causes JAK2 hyperphosphorylation and the consequent activation. (**B**) CBL is a well-known regulator of EGFR signaling, being able to induce EGFR ubiquitination and degradation. CBL binding can be mediated also by other adaptor proteins such as Grb2 and CIN85. (**C**) CBL activity also has a high influence on the PI3K/AKT signaling cascade. Through the interaction with LYN, it promotes the recruitment of PIK3R1 and the subsequent PI3K phosphorylation and activation.

As previously seen, diverse functions are mediated by the different CBL domains that interact with many protein targets, giving rise to a complex signaling network (Figure 2).

The TKB domain that mediates the recognition of phosphorylated tyrosine on activated RTKs, and the RF domain that recruits ubiquitin-conjugating enzymes, are primarily responsible for CBL ubiquitin-protein ligases function that controls ubiquitylation and downregulation of RTKs [13]. CBL has been reported to play a role in different well-known cell pathways, many of which are related to hematopoietic development, immunology and cancer onset and progression. Its double role as an E3 ubiquitin ligase and multi-adaptor protein, which is implicated in positive regulatory functions in signaling transduction, confers a gain of function mechanism in *CBL* mutant cells, associated with the disruption of E3 ubiquitin ligase activity. The gain of function phenotype is partly lost when there is co-occurrent presence of either the wild-type *CBL* allele or co-transduction of wild-type *CBL*. Indeed, this phenotype becomes apparent in the *CBL*-null background, which supports the observation that *CBL* mutations are often found in a homozygous state with loss of the wild-type allele. *CBL* mutations are often missense changes at highly conserved amino-acid positions within the linker and RF domains, which in turn leads to amino acid deletions within these domains. Although the E3 ubiquitin ligase activity primarily depends on the RF domain, the intact linker sequence is also considered structurally essential for an efficient ubiquitinylation to occur [14].

### 3.1. CBL and JAK2 Signaling

JAK2 is a tyrosine kinase implicated in the signaling of type II cytokine receptor family, including interferon, erythropoietin, granulocyte-macrophage colony-stimulating factor (GM-CSF) and many types of interleukins [15]. Its role is mainly related to the hematopoietic compartment, where it is involved in hematopoiesis and in immune system regulation. JAK2 function is regulated through the control of its phosphorylation status. CBL has been proven to promote GM-CSF-induced JAK2 activation and signaling, promoting its full phosphorylation after CBL-mediated ubiquitination at K970 residue [16]. On the other hand, JAK2 poly-ubiquitination occurs through CBL via the adaptor protein LNK/SH2B3, increasing its proteasomal degradation [17,18]. The CBL–LNK–JAK2 signaling complex has indeed been proved to regulate JAK2 ubiquitination, stability and activity.

### 3.2. CBL and EGFR–CBL–CIN85 Axis

CBL is one of the main regulators of the RTK EGFR [10], whose ubiquitination and consequent downregulation are strictly linked to CBL RING and TKB domain functions. The interaction between CBL and EGFR can also be mediated by other interactors such as the adaptor proteins Grb2 and CIN85. CIN85 contains a SH3 domain that is able to bind CBL promoting RTKs trafficking to endosomes prior to their lysosomal degradation [12]. In normal conditions, CIN85 binds CBL only under growth factor stimulation, while CBL mutants are able to interact with CIN85 even without any stimuli causing the deregulation of EGFR–CBL–CIN85 signaling and promoting oncogenic transformation. A strong demonstration of the acquisition of gain-of-function properties by mutant CBL is that the disruption of interactions between EGFR or CIN85 and mutant CBL reduces the oncogenic transformation.

### 3.3. CBL and PI3K/AKT/LYN Interaction

Another signaling axis playing a significant role in the gain-of-function phenotype conferred by *CBL* mutations is CBL–LYN–phosphatidylinositol 3-kinase (PI3K). *CBL* mutations have been reported to increase the expression levels of the PI3K/AKT pathway promoting tumoral onset [19,20]. This effect has been demonstrated to be strictly linked with the interaction with the LYN protein. In CBL mutant cells, increased LYN activation and interaction with mutant *CBL* enhance CBL phosphorylation, PI3K regulatory subunit 1 (PIK3R1) recruitment and downstream PI3K/AKT signaling activation [21].

## 4. CBL in Human Malignancies

### 4.1. CBL in JMML

The role of *CBL* as a cancer-predisposing gene was first identified in myeloid neoplasms, particularly in juvenile myelomonocytic leukemia (JMML) [22]. JMML is a rare clonal hematopoietic disorder that typically affects infants and young children, with a median age at presentation of 2 years [23]. It is characterized by alterations in the RAS pathway and about 90% of patients harbor a mutation in one of five genes (*PTPN11*, *NRAS*, *KRAS*, *NF1* and *CBL*) [24]. Clinically, JMML results in anemia, leukocytosis, thrombocytopenia and the infiltration of monocytic and granulocytic cells in different organs, chiefly the spleen.

Homozygous *CBL* mutations have been found in around 10% to 15% of all JMML cases [25,26]. Intriguingly, most of these children have a heterozygous germline CBL mutation. Leukemia development is associated with loss of heterozygosity (LOH) occurring, in the majority of cases, via a uniparental disomy resulting in 11q isodisomy in hematopoietic stem cells [27,28,29,30,31,32]. *CBL* mutations are mainly located throughout the linker and RF domains encoded by exons 8 and 9, especially in correspondence with Y371C [26,28], and consist of missense mutations or splice site variants [33]. The end result is the expression of a CBL protein with defective E3 ligase activity that constitutively activates the RAS pathway [31]. CBL mutated JMML presents low DNA methylation levels [34] and secondary genetic alterations are not commonly found [29,35]. The absence of other concomitant RAS pathway-associated mutations may indicate that *CBL* mutations play a pivotal role in deregulating this key pathway in JMML pathogenesis. Notably, clinical features and methylation profiling are not able to distinguish JMML with *CBL* germline mutations from patients with only somatic mutations [29].

This subgroup of JMML is associated with a relatively good prognosis. Most patients have a self-limiting indolent clinical course with spontaneous regression of the myeloproliferative disease without any therapy despite the persistence of LOH and clonal hematopoiesis [28,36,37]. Some children instead present an aggressive disease characterized by severe splenomegaly and platelet-transfusion dependency [28,38,39]. Therefore, a strict clinical follow-up without therapeutic intervention is recommended immediately after diagnosis (watch and wait strategy), and allo-HSCT is performed only if chromosomal aberrations occur or in case of disease progression [39]. If necessary, the choice of bridge to transplant therapy remains uncertain [39]. Interestingly, in a recent trial, the hypomethylating agent azacitidine demonstrated to provide a relevant clinical benefit in newly diagnosed JMML prior to HSCT, but patients with CBL mutations were excluded [40]. 

Spontaneous regression of the myeloproliferative disease sometimes occurs despite the persistence of LOH and clonal hematopoiesis [28,36,37]. Regardless, a high rate of stable mixed chimerism is observed after allo-HSCT [39,41]. Recently, a case of reversion of LOH after allo-HSCT with primary graft failure was reported in a child with CBL-related JMML. The patient experienced autologous hematopoietic recovery, but regained heterozygous mutational status through reversion of LOH and was disease-free at the end of the follow-up, indicating that hematopoietic reconstitution occurred within cells that did not harbor the JMML-causing homozygous mutation [42].

### 4.2. CBL in Hematological Neoplasms

Homozygous mutations in *CBL* have been found in a wide variety of myeloid neoplasms other than JMML. *CBL* mutations are frequent in chronic myelomonocytic leukemia (5–13% of patients) [43,44] and acute myeloid leukemia (AML), particularly forms secondary to myelodysplastic syndromes (9% of cases), suggesting that *CBL* mutations may contribute to leukemia development [9,43]. Furthermore, *CBL* mutations could also be found in myelodysplastic syndromes, chronic myelogenous leukemia and other chronic myeloproliferative diseases [9,43,44]. Similar to what has been described in JMML, the majority of patients show missense mutations clustering within the linker region and the RF domain [9]. Less frequently, exon 8 deletions have been reported [9,45]. Interestingly, most missense mutations are homozygous, while exon 8 deletions are usually heterozygous [9]. Although in vitro studies demonstrated that expression of *CBL* exon 8 and/or exon 9 deletions in a *CBL* wild-type context shows a transforming phenotype, in the AML patients this alteration was found only in concomitance with other aberrations, particularly CBF leukemia [46]. It is therefore possible to speculate that *CBL* mutations may cooperate in the pathogenesis of this form of AML [45,46]. Moreover, the majority of *CBL* mutations in myeloid neoplasms have been reported in the absence of RAS or PTPN11 mutations, highlighting the mutual exclusivity of *CBL* mutations and other RAS pathway alterations [9]. The prognostic impact of *CBL* mutations in myeloid malignancies is still controversial [9,44,47,48]. To date, no clear clinical association has been established, and this should be a focus of research in future. Despite somatic mutations in *CBL* being found in approximately 5% of myeloid neoplasms [9], leukemia other than JMML have not been reported frequently in patients with germline *CBL* mutations. To date, only two reports exist in the literature of AML developing in patients with CBL syndrome. Becker et al. described a case of AML in an adult patient with a heterozygous de novo germline mutation in *CBL* codon D390 located in the RF domain [49]. Somatic leukemia cells presented with homozygous *CBL* mutations resulting from copy-neutral LOH and an additional chromosomal gain at 11q. Additional mutations were found in the AML bone marrow, including inv(16) (p13q22) [49]. Interestingly, the bone marrow maintained *CBL* LOH even during complete remission and with normal blood counts, similar to what has been described in JMML [28,36,37]. In another report, Ali et al. addressed the case of an adult patient with missense *CBL* germline familial mutation in exon 8 developing AML. Leukemia arose due to copy-neutral LOH occurring through uniparental disomy. After induction chemotherapy, morphological remission was achieved, but atypical monocytosis and homozygous CBL mutation persisted in the absence of the previously detected AML-associated mutations and chromosomal alterations. After the consolidation course, the bone marrow still presented the same reported morphological and genetic features. The clinicians therefore suspected an underlying chronic myelomonocytic leukemia or pre-neoplastic monocytic expansion, and performed haploidentical allo-HSCT from her non-CBL-carrying son, achieving complete chimerism and complete remission [50]. A homozygous somatic mutation in *CBL* (p.C381R) associated with an 11q-acquired uniparental disomy was found in one patient with T-ALL but without evidence of germline predisposition [51]. Functional analysis revealed activation of the RAS and ERK pathways, whereas *CBL* mutations downregulated the NOTCH1 signaling pathway. ERK activation in cell-expressing p.C381R decreased with increasing wild-type CBL expression, highlighting the pathogenic importance of the wild-type *CBL* allele loss [51]. Mutations in *CBL* have also been identified in two infant ALL patients with MLL gene rearrangements [52] and in three children with B precursor ALL [53]. In the latter patients, small deletions affecting the intron/exon boundaries of exon 8 occurred, leading to the skipping of exon 8 and abolishing E3 ligase function. Moreover, constitutive activation of the RAS pathway and sensitivity to MEK inhibitors was found in mutated samples [53].

### 4.3. CBL in Other Malignancies

**Figure 3:**
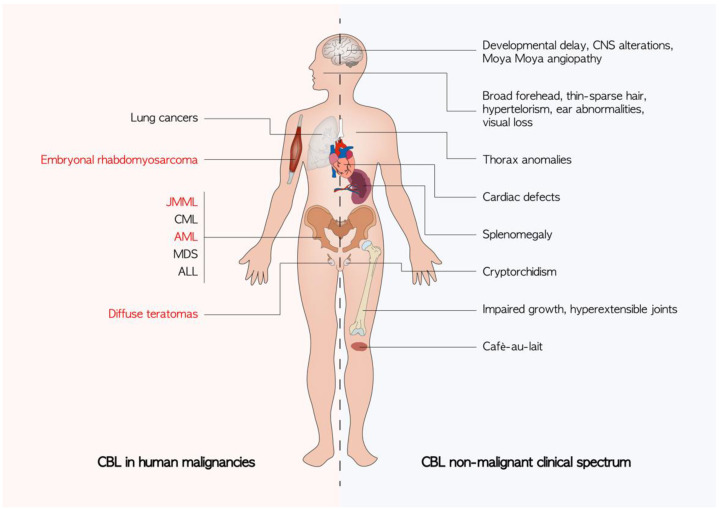
Known clinical manifestations of *CBL* mutations. The non-malignant phenotypes are an expression of germline *CBL* mutations. Cancers described in patients harboring a germline CBL mutation are highlighted in red. ALL: acute lymphoblastic leukemia; AML: acute myeloid leukemia; CML: chronic myelomonocytic leukemia; CNS: central nervous system; JMML: juvenile myelomonocytic leukemia; MDS: myelodisplastic syndrome.

The role of CBL in oncogenesis seems to not be limited to hematological malignancies. Somatic *CBL* mutations have been reported in lung cancers [54,55] while diffuse teratomas and embryonal rhabdomyosarcoma have been observed in patients with germline CBL mutations resulting in LOH in the CBL locus [56,57]. Daniels et al. recently catalogued the mutational spectrum found in various tumors of the three genes: *CBL*, *CBL*-b, and *CBL*-c in the TCGA database [58]. The mechanism by which CBL contributes to the pathogenesis of solid tumors has not been completely clear. Other than the already discussed effect on the RAS pathway, a fascinating hypothesis is to consider the role of CBL in tumor-mediated angiogenesis [59]. Tumors of c-*CBL* knockout mice indeed exhibit increased growth and higher angiogenesis compared with wild-type mice [60] (Figure 3).

## 5. CBL in Non-Malignant Clinical Spectrum

### 5.1. CBL Syndrome

In the last few years, the role of CBL in non-malignant phenotype has been progressively described. CBL genetic mutations can lead to a complex multisystemic syndrome characterized by variable phenotypic expression and expressivity known as “CBL syndrome” (OMIM #165360). It is a Noonan syndrome (NS)-like disorder that can be included in the vast field of RASopathies, a group of genetic entities caused by germline mutations in genes of the Ras/MAPK cellular pathway [61]. *CBL* mutations are thought to account for <1% of patients with clinical features resembling NS. To date, the increasing application of next generation sequencing (NGS) is revealing *CBL* germ-line mutations in 1–6% of patients who received a clinical diagnosis of NS or NS-like disorders in the absence of mutations in the known causative genes (e.g., *PTPN11, SOS1, KRAS, NRAS*) [62,63,64]. While most of the reported patients carried heterozygous CBL mutations, various clinical findings have also been described in patients with homozygous CBL mutations not developing JMML, as discussed below in detail.

The first reports of CBL syndrome arose from cohorts of children with *CBL*-mutated JMML initially described in the last decade [31]. In a cohort of 21 children diagnosed with homozygous *CBL*-mutated JMML, Niemeyer and colleagues identified 17 patients who harbored heterozygous *CBL* mutations in normal tissues [31]. In 13 children, autosomal inheritance was confirmed from parents’ genetic analysis. Dysmorphic facial features reminiscent of NS with broad forehead, hypertelorism and ear abnormalities associated with developmental delay, cryptorchidism and impaired growth were reported. Five patients presented juvenile xantogranuloma. *CBL* mutations affected the RF domain and Y371 was the most common mutation site. Functional studies confirmed an impaired ubiquitylation activity of heterozygous CBL-mutated proteins with defective E3 ligase activity that constitutively activate the Ras pathway [31]. Other authors confirmed these similar findings [26,32]. In the same period, Martinelli et al. analyzed the DNA samples of 365 patients who presented a phenotype consistent with RASopathy without a known causative mutation [62]. Six patients harbored four different heterozygous mutations in the *CBL* gene. The four mutations affected residues located within the RF domain or the adjacent linker domain, critical for the E3-ubiquitin ligase function. Similar to the other studies [31], in vitro analysis showed that these mutations resulted in impaired ubiquitylation and degradation of cell-surface receptor (e.g., EGF receptor) and dysregulating intracellular signaling through RAS pathway alteration. The clinical phenotype was highly variable and only one patient fulfilled diagnostic criteria for NS [65]. In addition to dysmorphisms and developmental delay, patients showed ectodermal and musculoskeletal features, particularly cafè-au-lait spots, thin-sparse hair, hyperextensible joints and thorax anomalies. Three children exhibited cardiac defects and none of the patients developed hematologic malignancies [62]. In a recent publication by the same group, five patients with five different pathological variants were identified in a large cohort of 349 patients exhibiting NS-like features, showing characteristic dysmorphic facial features and developmental delay together with neurosensorial alteration resulting in hearing or visual loss and cardiac defects, particularly pulmonary valve stenosis [66]. It has also been reported in a wide range of central nervous system alterations, including delayed myelination, corpus callosum abnormalities, cerebellar vermis hypoplasia, Arnold–Chiari malformation type I, mild left cerebral atrophy, widened cisterna magna and particularly moyamoya disease [62,66]. 

### 5.2. Vascular Pathology

**Figure 4:**
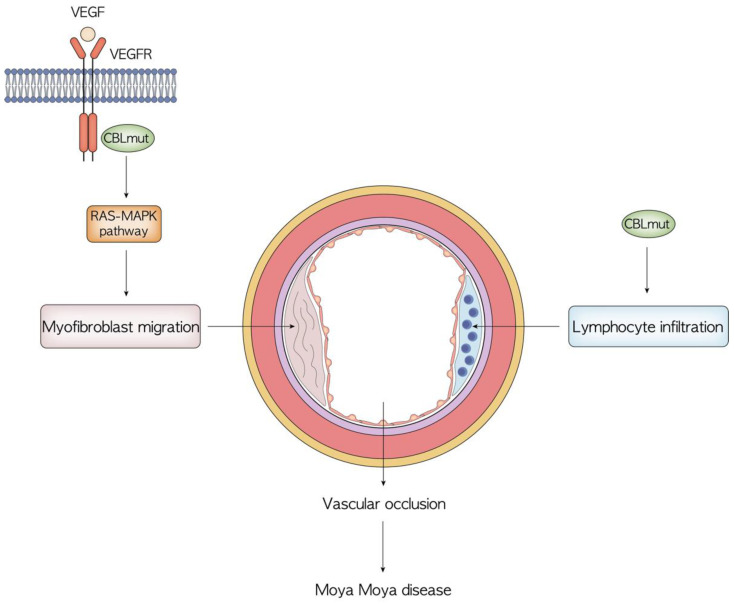
Overview of the possible pathogenesis of moyamoya disease in patients with germline CBL mutations. The role of *CBL* mutations in the genesis of vascular anomalies remains to be fully uncovered, particularly regarding the unknown mechanism that drives lymphocyte infiltration.

A small proportion of patients in the cohort described by Niemeyer et al. [31] presented vascular disorders, including Takayasu arteritis. Other reports later described vasculopathies primarily affecting cerebral blood vessels, such as moyamoya disease in patients with germline *CBL* mutations [37,67]. Recently, heterozygous somatic CBL mutations have also been reported in patients with cerebral arteriopathy of unknown origin [68,69]. Moyamoya disease is a cerebral vasculopathy characterized by a progressive stenosis of the terminal internal carotid arteries with subsequent development of abnormal collateral vessels, leading to ischemic and hemorrhagic stroke in both children and adults. The term moyamoya syndrome is used for patients with characteristic vasculopathy and specific associated genetic conditions [70]. The role of *CBL* mutations in the genesis of vascular anomalies remains to be fully uncovered. Studies on animal models revealed that Cbl-knockout in mice T and B cells causes lymphocyte vascular infiltration with vascular damage [71,72]. Moreover, impaired Cbl-mediated signaling through the RAS-MAPK pathway leads to uncontrolled VEGF stimulation promoting angiogenesis. Myofibroblast migration and proliferation seems to also be enhanced, possibly determining vascular occlusion. Interestingly, these effects are corrected with MAPK pathway inhibitor therapy [71,72]. No case of vasculitis has been reported in patients who received transplantation, suggesting a key role of lymphocyte dysregulation in the genesis of vascular pathology [31] (Figure 4).

Moreover, children transplanted for JMML frequently experienced stable mixed chimerism that apparently results sufficient to restore an adequate immune system control [31,61]. One patient transplanted for JMML also showed correction of the associated moyamoya disease without progression of the vascular disease after transplant [67]. This evidence suggests the presence of a pathological role of mutated *CBL* for moyamoya and vasculitis, and thus HSCT might represent an efficacious therapy for both.

### 5.3. Immunological and Hematological Manifestations

Among reported patients with *CBL* germline mutations, a large part experienced variable immunological and hematological features. Splenomegaly is frequent in patients with *CBL* mutations even in the absence of JMML. Coe et al. reported splenomegaly as the presenting feature of CBL syndrome in a child but pointed out that it was not possible to rule out an undetected episode of JMML during infancy with spontaneous resolution but persistent splenomegaly later in life [73]. In a large family carrying Y371C *CBL* mutation, one member presented hepatosplenomegaly with granulocytic hyperplasia of the bone marrow while two other presented monoclonal indolent gammopathy and monocytosis [74]. This family also pointed out the presence of *CBL* germline mutation without any clinical manifestations. Splenomegaly was also described in two patients with CBL syndrome before adult-onset AML. The authors hypothesized that an undiagnosed transient myeloproliferative disorder or JMML with spontaneous remission may have occurred during childhood [49,50]. Interestingly this latter patient also presented a history of autoimmunity, namely rheumatoid arthritis and immune thrombocytopenia [50]. Autoimmune uveitis was also reported in a patient with *CBL*-mutated JMML presenting an indolent course of the malignancy without any treatment [75]. Interestingly, uveitis was successfully treated with anti-TNF adalimumab [75]. Regarding immunological dysregulation in a large cohort of pediatric patients with Evans Syndrome (ES), a rare severe autoimmune disorder characterized by bilinear cytopenia, one patient exhibited a germline *CBL* mutation suggesting a role in the pathogenesis of the autoimmune phenotype [76]. As previously mentioned, specific immunological and hematological features have been reported in patients with homozygous mutations not developing JMML. Three patients with homozygous mutation derived by LOH all showed a clonal proliferation of homozygous CBL-mutated cells in peripheral blood. The homozygous mutation persisted in blood cells in long-term surveillance even after clinical resolution. These patients also presented splenomegaly and mild monocytosis associated with moyamoya diseases, neutrophil and monocyte proliferation and severe hemophagocytic syndrome secondary to EBV infection [37]. A recently described 1-year-old boy with homozygous *CBL* mutation with LOH in peripheral blood cells developed lymphoid proliferation with cytopenia, hepatosplenomegaly, lymphocytic lymphocytosis and B-cell expansion [77]. Recently, a patient with known moyamoya disorder, cryptorchidism, dysmorphic features, splenomegaly and thrombocytopenia who developed atypical hemolytic uremic syndrome was found to carry heterozygous mutation in *CBL*. He also displayed evidence of myeloproliferation and subclinical JMML with expansion of clones harboring homozygous mutation of *CBL* due to LOH across chromosome 11q [78].

### 5.4. Coagulative Disorders

Coagulative disorders were described in patients with *CBL* germline mutations and in two patients with moyamoya disease. The first presented transient alterations, namely prolonged prothrombin time and elevated kaolin clotting time (KCT) ratio, that progressively normalized at 10 years of age. The second was diagnosed of antiphospholipid syndrome (APL) with persistent elevated cardiolipin and anti-β2GP1 antibodies together with elevated KCT ratio. APL syndrome has not been previously described in CBL syndrome, but seems to represent a common finding in other RASopathies [79]. In addition, the case of a patient with adult-onset AML and CBL syndrome with an history of coagulopathy during childhood was reported, specifically low levels of FVII, X, XII and XIII [49].

### 5.5. Prenatal Manifestation of CBL Germline Mutations

Prenatal and neonatal manifestations of *CBL* germline mutations include hydrops fetalis, fetal pleural effusions and congenital hydro-chylothorax and have been reported in one study. Similar prenatal manifestations have been associated with other RASopathies, suggesting a role of *CBL* mutations in perturbing the lymphangiogenesis signaling [80]. For instance, primary lymphedema has been described in a patient with germline *CBL* mutation [57].

## 6. Therapeutic Potential of CBL Targeting

The ubiquitination–proteasomal system represents a fundamental cell process which has attracted intense attention as a potential drug target. Many preclinical and clinical trials are indeed underway to test a wide range of compounds for pathologies such as blood and solid cancers [81,82,83]. In this context, CBL represents an ideal candidate as it is widely expressed in lymphoid and myeloid cells. Nonetheless, its involvement in different, even contrasting, functions requires attention in evaluating potential solutions. Overall, the important role of CBL in positively regulating major cellular functions questioned how to achieve its activation as a therapeutic target. Its constitutively autoinhibited conformation requires a phosphorylation of Tyr371, which in turn suggests that by targeting kinases or phosphatases modulating this phosphorylation, CBL could be manageable as a drug target, maximizing the benefits while reducing off-targets. The targeting of downstream signaling cascades could be another intriguing option, including JAK/STAT, PI3K, as well as RAS/extracellular signal-regulated kinase (ERK) cascade [14]. Recently, Belizaire and colleagues [19] demonstrated that LYN inhibition by dasatinib effectively diminished the expansion of *CBL* mutant cell lines and primary CMML samples in vitro and in vivo. This potential of LYN-targeted therapies in patients with *CBL*-mutated myeloid disease was observed by Bunda et al. as well in a study on chemo-resistant *CBL* mutant JMML cells. Their results supported the potential benefit of combining LYN-PI3K/Akt signaling modulation with traditional chemotherapy agents in the management of this disease. Indeed, the pharmacologic inhibition of Src family kinases with PP2 or dasatinib was able to reverse the GM-CSF sensitivity of JMML cells [84] to attenuate the hyperphosphorylation of CBL (Y371H) and p85 recruitment, thus inhibiting the subsequent Akt phosphorylation and activation.

Because the oncogenic action of mutant CBL proteins depends on their intact binding to target kinases, inhibition of this binding would be a potential approach, especially when the inhibition is specifically directed to mutant CBL and CBL-c, but be saved for CBL-b. Recently, piceatannol, a naturally occurring phenol stilbenenoid, was shown to induce loss of the CBL family proteins including mutant CBL and a broad spectrum of tyrosine and serine-threonine kinases [85].

## 7. Conclusions and Future Directions

*CBL* germline mutations are responsible for a complex multisystemic disease with an increased risk for developing malignances, recently defined as CBL Syndrome. Even though the clinical manifestations have been progressively characterized in recent years, the complete clinical spectrum seems far from being entirely described. Further cases are needed to exactly define the whole spectrum of CBL mutations. A complete clinical and molecular characterization is highly awaited since this would have profound implication in patients’ clinical management. Expert opinion-based recommendations for patients with *CBL*-mutated JMML are already available [39], while those for CBL syndrome are still lacking. Patients with molecularly confirmed *CBL* germline mutations should receive cancer surveillance and a full screening to assess cardiac, vascular and hematological alterations. On the other hand, clinical indications for the investigation of *CBL* mutations in specific conditions, such as early-onset moyamoya disease should be provided. In conclusion, CBL may be the key to an intriguing network of molecular mechanisms in a wide clinical spectrum and might represent a promising therapeutic target.
